# The ARIMA model approach for the biofilm-forming capacity prediction of *Listeria monocytogenes* recovered from carcasses

**DOI:** 10.1186/s12917-024-03950-y

**Published:** 2024-03-27

**Authors:** Adalet Dishan, Mukaddes Barel, Serhat Hizlisoy, Recep Sinan Arslan, Harun Hizlisoy, Dursun Alp Gundog, Serhat Al, Zafer Gonulalan

**Affiliations:** 1 Faculty of Veterinary Medicine, Department of Food Hygiene and Technology, Yozgat Bozok University, Yozgat, Turkey; 2 Faculty of Veterinary Medicine, Department of Veterinary Public Health, Erciyes University, Kayseri, Turkey; 3 Faculty of Engineering and Architecture, Department of Computer Engineering, Kayseri University, Kayseri, Turkey

**Keywords:** ARIMA, Biofilm, *L. monocytogenes*, Prediction, Virulence

## Abstract

The present study aimed to predict the biofilm-formation ability of *L. monocytogenes* isolates obtained from cattle carcasses via the ARIMA model at different temperature parameters. The identification of *L. monocytogenes* obtained from carcass samples collected from slaughterhouses was determined by PCR. The biofilm-forming abilities of isolates were phenotypically determined by calculating the OD value and categorizing the ability via the microplate test. The presence of some virulence genes related to biofilm was revealed by QPCR to support the biofilm profile genotypically. Biofilm-formation of the isolates was evaluated at different temperature parameters (37 °C, 22 °C, 4 °C and − 20 °C). Estimated OD values were obtained with the ARIMA model by dividing them into eight different estimation groups. The prediction performance was determined by performance measurement metrics (ME, MAE, MSE, RMSE, MPE and MAPE). One week of incubation showed all isolates strongly formed biofilm at all controlled temperatures except − 20 °C. In terms of the metrics examined, the 3 days to 7 days forecast group has a reasonable prediction accuracy based on OD values occurring at 37 °C, 22 °C, and 4 °C. It was concluded that measurements at 22 °C had lower prediction accuracy compared to predictions from other temperatures. Overall, the best OD prediction accuracy belonged to the data obtained from biofilm formation at -20 °C. For all temperatures studied, especially after the 3 days to 7 days forecast group, there was a significant decrease in the error metrics and the forecast accuracy increased. When evaluating the best prediction group, the lowest RMSE at 37 °C (0.055), 22 °C (0.027) and 4 °C (0.024) belonged to the 15 days to 21 days group. For the OD predictions obtained at -20 °C, the 15 days to 21 days prediction group had also good performance (0.011) and the lowest RMSE belongs to the 7 days to 15 days group (0.007). In conclusion, this study will guide in using indicator parameters to evaluate biofilm forming ability to predict optimum temperature-time. The ARIMA models integrated with this study can be useful tools for industrial application and risk assessment studies using different parameters such as pH, NaCl concentration, and especially temperature applied during food processing and storage on the biofilm-formation ability of *L. monocytogenes.*

**Supplementary Information:**

The online version contains supplementary material available at 10.1186/s12917-024-03950-y.

## Introduction

*Listeria monocytogenes* remarks public health issues with serious infection tables such as septicemia, meningitis, meningoencephalitis in immunosuppressives, invasive infections in the newborn and elderly, and severe complications in pregnancy [1, 2]. It can be found broadly spread in the food processing environment as a ubiquitous bacterium with a durable growth profile and causes a significant burden and challenge for food safety [3]. The occurrence of microbial communities in the food facility leads to a constant microbial reservoir that forms a lasting source of contamination [4]. *L. monocytogenes* can establish biofilms on food surface materials utilized found in the typical processing plants such as stainless steel, polystyrene, polypropylene, glass, marble, and granite. Non-food contact surfaces, generally wet and related to grounds and drains, are also a concern due to the likelihood of *L. monocytogenes* [5]. Pathogen management at the slaughterhouse level is indispensable to obstructing the entrance of *L. monocytogenes* in meat cutting and processing facilities throughout the agri-food chain. Pathogen presence in the eventual meat product can result from contaminated carcasses [6]. Bacteria in biofilms are generally more resistant to standard cleaning and sanitizing operations, which may also induce the existance of bacteria [7]. Spoilage and/or pathogenic bacteria can be conveyed to the food product by direct contact or by biofilms detaching from non-food contact surfaces to food contact surfaces during operation [8]. The key approach to maintaining safe food production is monitoring possible biofilm formation at early stages in the food environments [3]. Notwithstanding various assays for the biofilm-forming potential of *L. monocytogenes* strains, the crystal violet assay is the most frequently used biofilm quantification microtiter plate method [9]. The high-yielded capability allows simultaneous testing of multiple *L. monocytogenes* strains beneath different conditions [5]. The outcomes acquired regarding absorbances obtained at specific wavelengths correspond to raw data revealed in the test system. While quantitative testing is mainly considered more dependable, conventional microbiological analyses are time-consuming, error-prone, and expensive [10]. Predictive microbiology which aims to develop mathematical equations for describing the behavior of microorganisms under various environmental factors, is assembling traditional microbiology knowledge with the disciplines of mathematics, statistics and present information and technological systems [11]. Autoregressive integrated moving average (ARIMA), time series models aiming to make predictions for the future with the help of observation values from past periods, are widely used in many fields such as medicine, engineering and finance [12–14]. ARIMA models, which assume a linear relationship between the data forming the series and reveal this linear relationship, can be successfully applied to the time series [15]. The current study aimed to predict the biofilm-formation ability of *L. monocytogenes* obtained from carcasses via the ARIMA using different temperatures and times.

## Method

**Fig. 1:**
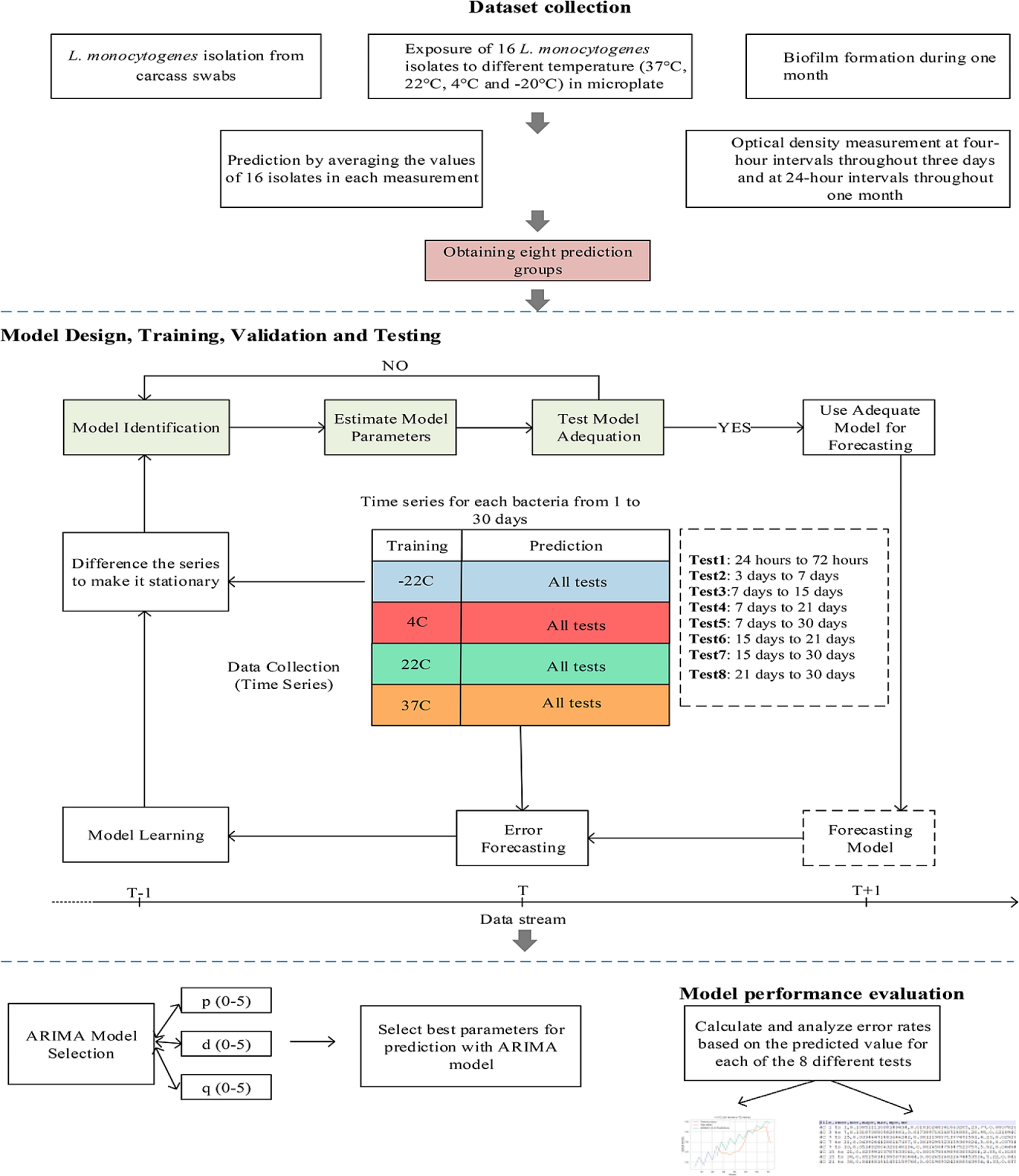
Data collection and methodology diagram of the prediction model

The data collection and prediction model diagram of this study is shown in Fig. 1.

### Sampling and isolation of *L. monocytogenes*

Four hundred carcass swab samples were collected from various four cattle slaughterhouses in Kayseri by visiting sixteen times between April and November 2021. At each visit, swap samples from the carcass (*n* = 25) were taken according to ISO 17,604 after the last washing of the carcass [16]. Selected four regions (chest, neck, buttock and posterior lateral hock) were sampled from an area of approximately 100 cm^2^ (total: 400 cm^2^) using a sterile square plastic template. The swabs were individually added to tubes containing a sterile transport solution, then transported to the laboratory in the cold chain within one hour and subjected to analysis. Isolation procedure was carried out following the ISO 11290-1:2017 [17]. Swab samples were enriched in Half Fraser broth (Merck, Germany) as primary enrichment. The inoculum were exposed for 48 h at 30ºC for incubation. Each sample’s culture (100 µL) was inoculated into 10 mL of Fraser broth and incubated at 37 ºC for 48 h. A loopful of inoculum was spread on Oxford Listeria Selective Agar and incubated for 48 h at 37 ºC. Suspicious colonies were purified in a blood agar and stored in cryoprotectant at -80 ºC.

### DNA extraction and polymerase chain reaction (PCR) amplification

Total genomic DNA isolation of the isolates was performed with the Instagene Genomic DNA Extraction kit (Bio-Rad, USA) following the kit protocol. For the identification of *L. monocytogenes*, the LM1 and LM2 primer pair reported by Border et al. (1990) was used [18]. DreamTaq Hot Start PCR (2x) Master Mix (Thermo Fisher, USA) was used in PCR analysis in accordance with the manufacturer’s instructions. In the confirmation of *Listeria* spp., after pre-denaturing for two minutes at 95 °C, 30 s at 95 °C, 30 s at 56 °C, 1 min (35 cycles) at 72 °C, and 10 min at 72 °C final elongation protocol was applied (ArkticTM Thermal Cycler; Thermo Fisher, USA). For the identification of *L. monocytogenes*, after pre-denaturation at 95 °C for two minutes, at 95 °C for 30 s, at 54 °C for 30 s, at 72 °C for 1 min (35 cycles) and at 72 °C for 10 min final extension protocol was performed (ArkticTM Thermal Cycler; Thermo Fisher, USA). Amplicons were loaded onto 1.5% agarose gel, subjected to electrophoresis at 120 volts for 45 min and visualized.

### Detection of virulence genes by quantitative-PCR (QPCR)

The presence of virulence genes *hly*, *sigB*, *plcA*, *plcB*, *inlA, inlB*, *inlC*, and *inlJ* was investigated using Sybergreen QPCR [19–21]. For this purpose, SYBR Green Master Mix (Bio-Rad) was used with each primer at a concentration of 10 pmol according to the manufacturer’s instructions. Real-time PCR analyzes were performed with the CFX96 Touch Real-Time PCR analyses (Bio-Rad). The samples’ detection rates and quantitative values were determined using amplification curves, melting curve analysis and Ct (dR) data. The description of all virulence primer pairs used for the present study is given in Supplementary Table 1.

### Biofilm production analysis

Biofilm production was measured by the conventional microbiological crystal violet test on cultured biofilms in microtiter plates using Stepanović et al. (2000) method in triple experiments [22]. Bacteria were grown overnight in brain heart infusion broth (BHI, Merck, Germany). A microtiter plate containing BHI was inoculated with overnight culture at a dilution 1:200 and incubated. The inoculums were subjected to a crystal violet assay for in vitro biofilm-formation at four-hour intervals throughout three days and at 24-hour intervals throughout one month in different temperatures (37 °C, 22 °C, 4 °C and − 20 °C). For analysis of biofilm production, the medium was removed from the wells and washed twice with sterile physiological saline. The remaining attached bacteria were fixed with 200 µL of 99% methanol per well, and after 15 min, plates were emptied and left to dry. After that, 1% crystal violet solution was added and incubated for 10 min. After washing three times with distilled water, the plates were air dried, and the dye bound to the adherent cells was resolubilized with 160 µL of 33% (v/v) glacial acetic acid per well. Absorbance was measured at 570 nm in an enzyme-linked immunosorbent assay reader (ELISA; Thermo Scientific, USA) reader. The original data assessment was made according to the following: OD ≤ ODc biofilm negative, ODc < OD ≤ 2X ODc weak biofilm, 2X ODc < OD ≤ 4X ODc moderate biofilm, and OD > 4x ODc strong biofilm formation [22]. Sterile BHI broth was used as blank control. All measurements were performed in triplicate.

### Original data assessment

Graphics with error bars and heat map showing the original data evaluation were obtained from Microsoft Excel.

### Biofilm OD prediction grouping

The data in the study were collected in eight prediction groups (Table 1).

**Table 1:** Description of forecast groups

Grouping Name	Estimation Conditions
24 to 72 h	72nd hour OD by the measurement data at four hours intervals in 24 h
3 days to 7 days	day 7 OD by 24, 48, 72nd-hour measurement data
7 days to 15 days	day 15 OD by measurement data at 24-hour intervals between days 1–7
7 days to 21 days	day 21 OD by measurement data at 24-hour intervals between days 1–7
7 days to 30 days	day 30 OD by measurement data at 24-hour intervals between days 1–7
15 days to 21 days	day 21 OD by measurement data at 24-hour intervals between days 1–15
15 days to 30 days	day 30 OD by measurement data at 24-hour intervals between days 1–15
21 days to 30 days	day 30 OD by measurement data at 24-hour intervals between days 1–21

### Autoregressive integrated moving average (ARIMA)

The classical Box-Jenkins Models performed the time series forecasting related to biofilm OD prediction grouping. In the ARIMA (p, d, q) model, the p-value is the degree of autoregression parameter; The d-value represents the number of differentiating operations; The q-value is the degree of the moving average parameter; and the t-value represents the time. The general expression of the ARIMA (p, d, q) model can be shown as follows [23, 24]:

$$\eqalign{{w_t} & = {\theta _1}{w_{t - 1}} + {\theta _2}{w_{t - 2}} +... + {\theta _3}{w_{t - p}} \cr & + {a_t} - {\theta _1}{a_{t - 1}} - {\theta _1}{a_{t - 2}} - \ldots - {\theta _q}{a_{t - q}} \cr}$$

The moving average parameters (θ) are defined as negative in the equation following the Box-Jenkins convention. If the first differences make the series stationary, the d = 1 difference equation, if the second differences make the series stationary, the d = 2 difference equation will emerge.

$$\eqalign{ d & = 0:\;{w_t} = {W_t} \cr d & = 1:\;{w_t} = {W_t} - {W_{t - 1}} \cr d & = 2:\;{w_t} = ({W_t} - {W_{t - 1}}) - ({W_{t - 1}} - {W_{t - 2}}) \cr}$$

Development of the model was implemented by Python version 3.10.6 and matplotlib, pandas, seaborn, numpy, imblearn, statsmodels, and xlsxwriter libraries.

### Model performance evaluation

The ARIMA model performance was measured by metrics including mean absolute value (MAE), mean absolute value error (MAPE), mean square error (RMSE) and mean square error (MSE) [25]. According to the evaluation, the small difference between the predictions made for the test set prepared for the proposed model and the actual values showed that the error measurement values were at a minimum level. Error values were calculated with the metrics package in the sklearn library. These metrics are expressed below.

$$\eqalign{ MAE & = {1 \over n}\sum\limits_{i = 1}^n {\left| {{y_i} - {{y^{\prime}}_i}} \right|} \cr MSE & = {{\sum\nolimits_{i = 1}^n {{{\left| {{y_i} - {{y^{\prime}}_i}} \right|}^2}} } \over n} \cr MAPE & = {1 \over n}\sum\limits_{i = 1}^n {{{\left| {{y_i} - {{y^{\prime}}_i}} \right|} \over {{y_i}}}} \times 100 \cr RMSE & = \sqrt {{1 \over n}\sum\limits_{i = 1}^n {{{\left| {{y_i} - {{y^{\prime}}_i}} \right|}^2}} } \cr}$$

### Validation

The work was basically in 2 stages. The 1st stage is the collection of data for 8 different groups and the 2nd stage was to carry out the model design, training, validation and testing processes. Validation was a critical processes for ARIMA modeling. The most basic approach used to do this was to divide the original data into training and test sets and compare the predicted values with the actual values using error measurements for the test set. The data was trained in a loop for each test group, validation was performed and tested, and the error value was calculated. This process continued by moving on the data step by step. The walk-forward validation process was carried out and the proposed model and the model with the lowest error value were revealed. As a result, the approach of determining and validating the best prediction model for each group and using the most successful model in the testing phase was adopted (Fig. 1).

### Statistical analysis

Based on the original mean values, the statistical significance of time at each temperature on biofilm formation was determined by analysis of variance. Group comparisons were conducted with the Tukey HSD multiple comparison test. Statistical programming with R was used (www.r-project.org/). The significance level was determined as *p* < 0.05.

## Results

### Occurrence of *L. monocytogenes* and detection of virulence genes by qPCR

Of 400 samples, 16 (4%) isolates were found positive for *L. monocytogenes*. 14 (87.5%) of the positive isolates were deemed suitable for biofilm formation prediction. Virulence profiles of 14 isolates used in the study to estimate biofilm formation ability are shown in Supplementary Table 2. While no isolates were found to harbor the *pclA* gene, the distribution of *sigB*, *inlA*, *inlJ, plcB*, *inlC*, *inlB* and *hlyA* virulence target genes among these 14 isolates was determined as 71.4%, 71.4%, 35.7%, 28.4%, 21.4%, 14.2% and 14.2%, respectively.

### Biofilm-formation profiles

#### Original value assessment

**Fig. 2:**
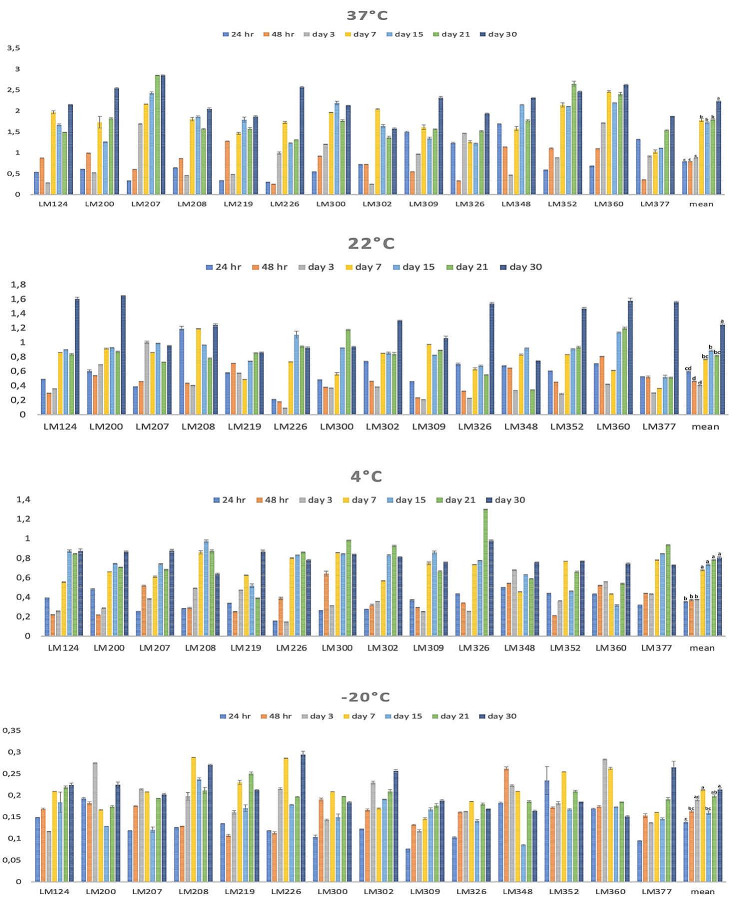
Biofilm formation profile of 14 *L. monocytogenes* isolates in the study. **a**, **b**, **c**: Means shown with different exponent letters are statistically significant (p<0.05). x-axis: isolate codes, y-axis: OD values

**Fig. 3:**
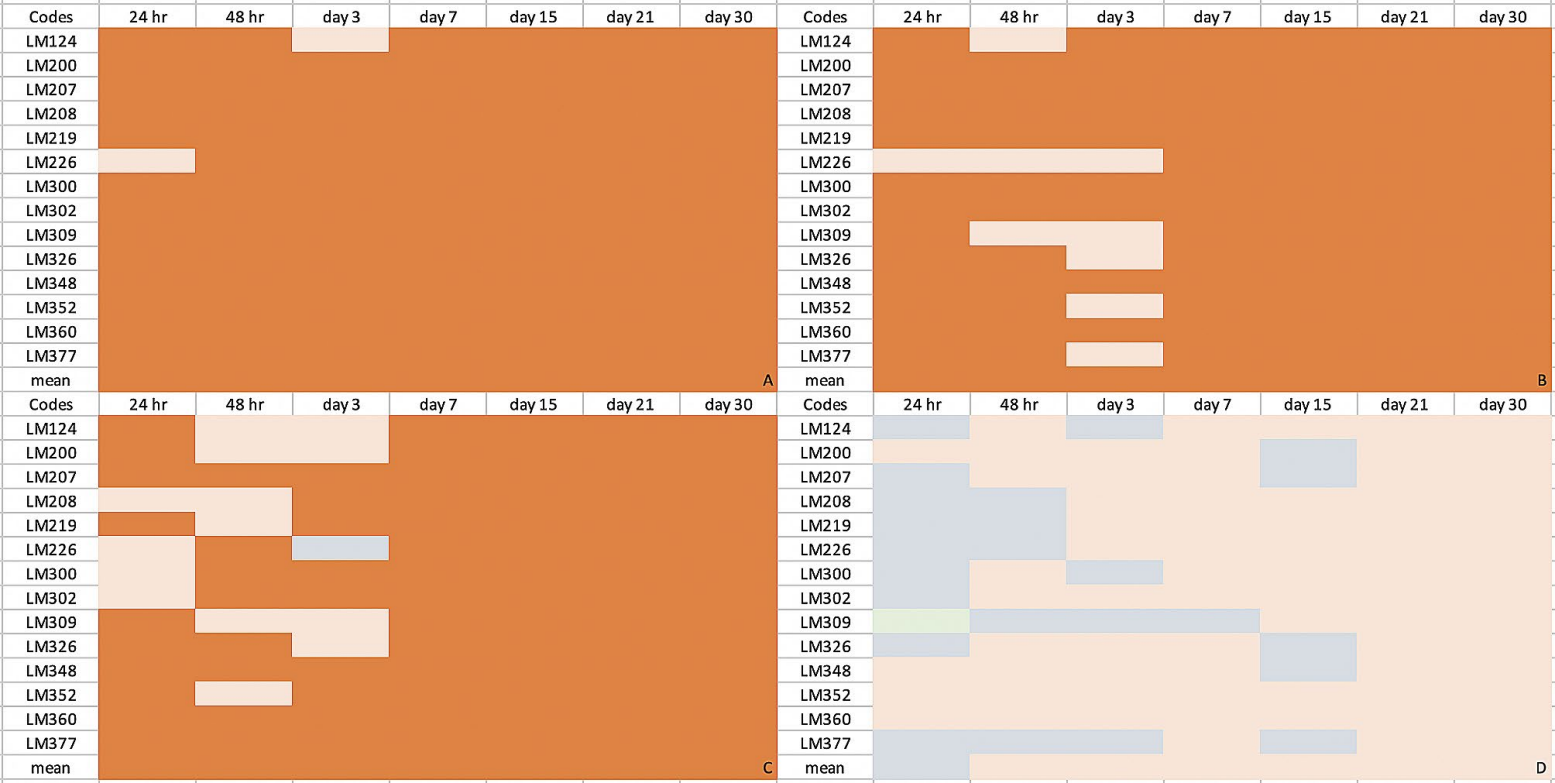
Heat map showing the biofilm formation ability of the 14 *L. monocytogenes* isolates at 37 °C (**A**), 22 °C (**B**), 4 °C (**C**) and − 20 °C (**D**) (Orange: strong biofilm former, salmon color: moderate biofilm former, grey: weak biofilm former, green: biofilm negative)

Figure 2 displays the original OD values of *L. monocytogenes* isolates at 37 °C, 22 °C, 4 °C and − 20 °C; Fig. 3 delivers heat maps showing the ability to form biofilms. The change over time in the average OD values obtained from biofilm formation at each temperature was found to be statistically significant (*p* < 0.05). Except for one of the *L. monocytogenes* isolates kept at 37 °C, it was observed that all isolates had strong biofilm properties from the 24th hour. All isolates kept at 22 °C and 4 °C from the first week showed strong biofilm-formation properties. All isolates kept at -20 °C after the 15th day were moderated.

### Comparison of the original value and the predicted value at different temperature parameters

**Fig. 4:**
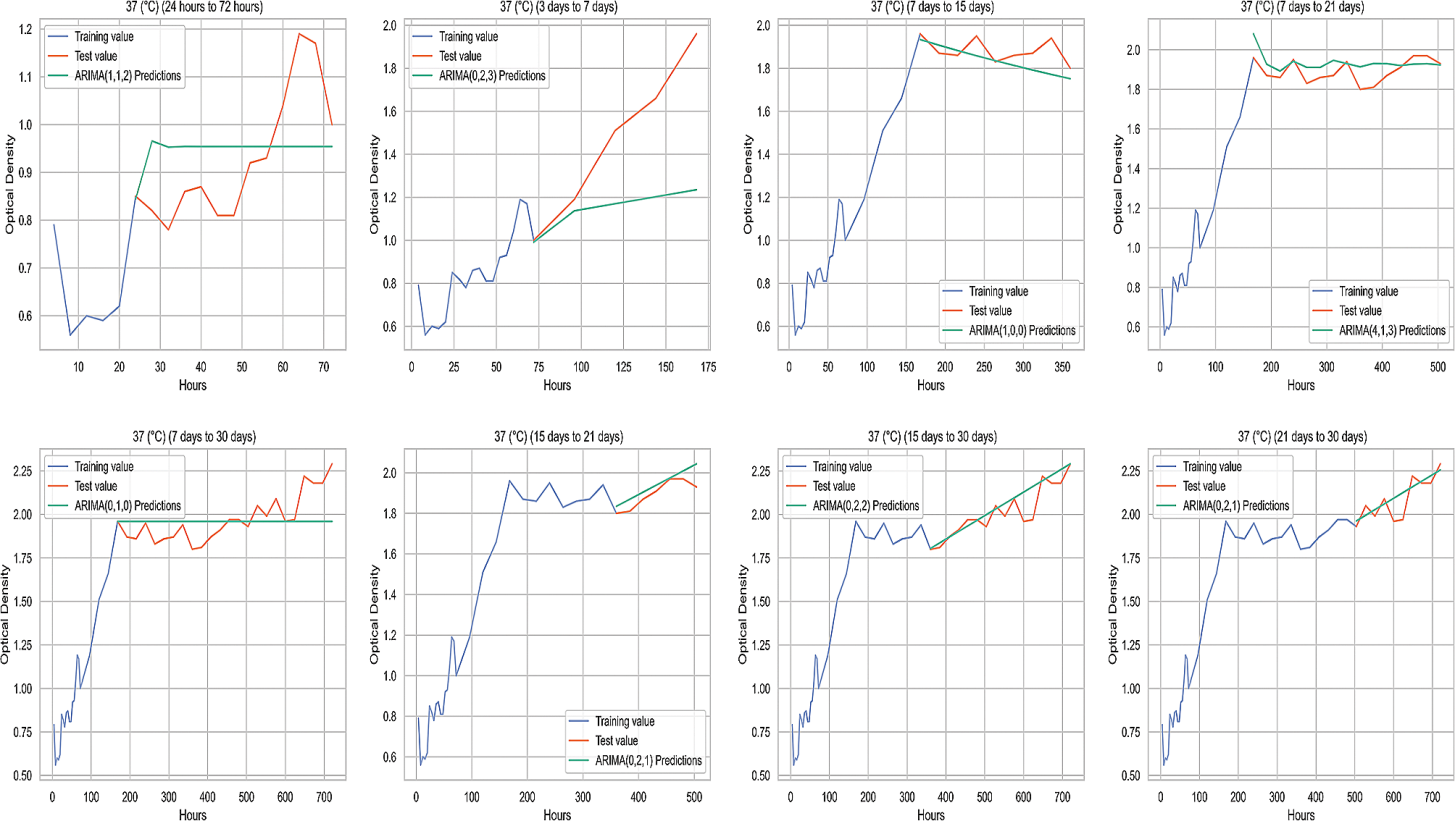
Comparison of the original value and the predicted value at 37 °C

**Fig. 5:**
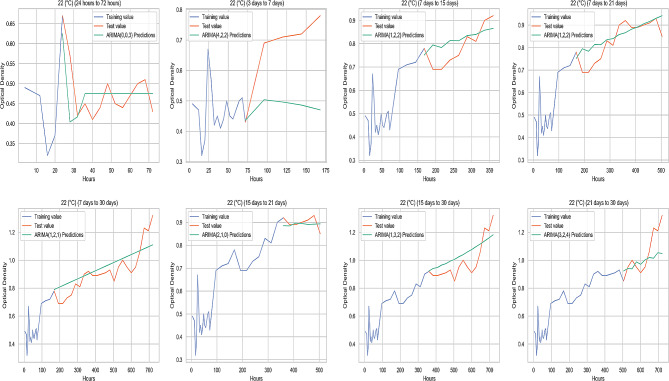
Comparison of the original value and the predicted value at 22 °C

**Fig. 6:**
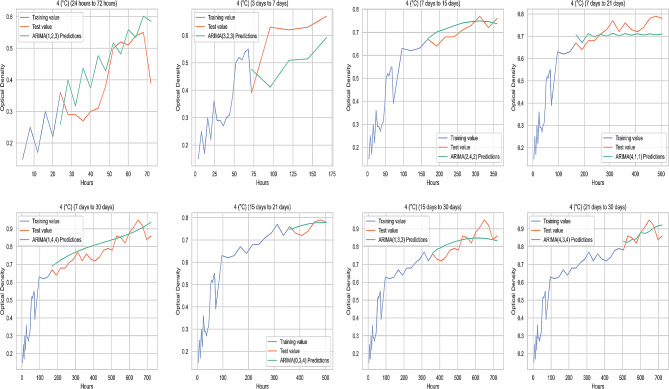
Comparison of the original value and the predicted value at 4 °C

**Fig. 7:**
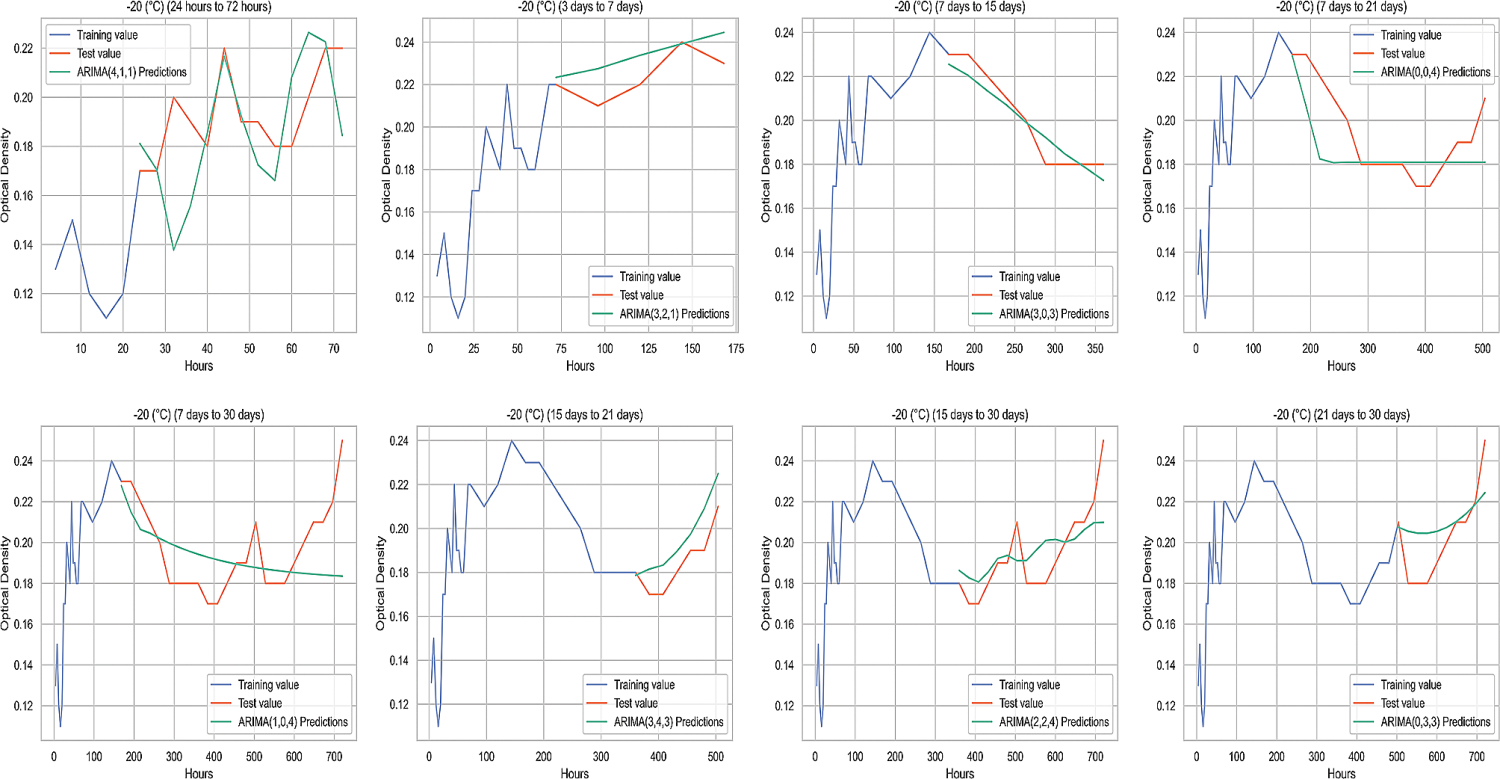
Comparison of the original value and the predicted value at -20 °C

The graphichs containing the average original values (OD) and ARIMA prediction values (OD) of biofilm-forming isolates at 37 °C, 22 °C, 4 °C, and − 20 °C obtained from the study are illustrated in Figs. 4, 5 and 6, and Fig. 7, respectively.

### The ARIMA model performance

The values of the error metrics showing the ARIMA model performance obtained from the average of the measurements at 37 °C, 22 °C, 4 °C, and − 20 °C are given in the Tables 2, 3 and 4, and Table 5, respectively.

**Table 2:** Error values of the average estimated values of the measurements obtained from biofilm formation at 37 °C for 14 isolates

Prediction groups	RMSE	MSE	MAPE	MAE	MPE	ME
24 to 72 h	0.131	0.017	11.870	0.110	-0.039	-0.020
3 days to 7 days	0.413	0.171	18.480	0.317	0.185	0.317
7 days to 15 days	0.075	0.006	3.070	0.058	0.023	0.044
7 days to 21 days	0.068	0.005	2.960	0.055	-0.022	-0.041
7 days to 30 days	0.131	0.017	4.800	0.097	0.002	0.012
15 days to 21 days	0.055	0.003	2.380	0.045	-0.024	-0.045
15 days to 30 days	0.074	0.005	2.370	0.047	-0.018	-0.036
21 days to 30 days	0.074	0.006	2.910	0.059	-0.012	-0.023

**Table 3:** Error values of the average estimated values of the measurements obtained from biofilm formation at 22 °C for 14 isolates

Prediction groups	RMSE	MSE	MAPE	MAE	MPE	ME
24 to 72 h	0.057	0.003	8.290	0.041	-0.001	0.005
3 days to 7 days	0.215	0.046	26.160	0.190	0.256	0.188
7 days to 15 days	0.064	0.004	7.450	0.056	-0.043	-0.028
7 days to 21 days	0.055	0.003	5.350	0.041	-0.033	-0.023
7 days to 30 days	0.090	0.008	8.040	0.073	-0.046	-0.031
15 days to 21 days	0.027	0.001	2.430	0.022	0.008	0.008
15 days to 30 days	0.095	0.009	8.540	0.083	-0.057	-0.048
21 days to 30 days	0.128	0.016	8.680	0.098	0.038	0.053

**Table 4:** Error values of the average estimated values of the measurements obtained from biofilm formation at 4 °C for 14 isolates

Prediction groups	RMSE	MSE	MAPE	MAE	MPE	ME
24 to 72 h	0.101	0.010	23.770	0.081	-0.182	-0.059
3 days to 7 days	0.132	0.017	20.950	0.122	0.121	0.087
7 days to 15 days	0.033	0.001	4.230	0.029	-0.030	-0.0197
7 days to 21 days	0.044	0.002	5.060	0.038	0.027	0.022
7 days to 30 days	0.051	0.003	5.920	0.045	-0.049	-0.035
15 days to 21 days	0.024	0.001	2.550	0.019	-0.015	-0.011
15 days to 30 days	0.052	0.003	5.220	0.043	-0.008	-0.002
21 days to 30 days	0.045	0.002	4.330	0.037	-0.008	-0.005

**Table 5:** Error values of the average estimated values of the measurements obtained from biofilm formation at -20 °C for 14 isolates

Prediction groups	RMSE	MSE	MAPE	MAE	MPE	ME
24 to 72 h	0.026	0.001	9.620	0.019	0.033	0.007
3 days to 7 days	0.012	0.000	4.550	0.010	-0.044	-0.010
7 days to 15 days	0.007	0.000	2.800	0.006	0.007	0.002
7 days to 21 days	0.017	0.000	5.990	0.012	0.039	0.009
7 days to 30 days	0.021	0.000	7.770	0.016	0.010	0.004
15 days to 21 days	0.011	0.000	4.680	0.009	-0.005	0.000
15 days to 30 days	0.015	0.000	5.750	0.011	-0.013	-0.001
21 days to 30 days	0.017	0.000	6.730	0.013	-0.043	-0.007

## Discussion

*Listeria* is mostly present in complex biofilms in wild habitats as a microbial response [26]. Wieczorek et al. (2012) found that bovine carcasses were positive for *L. monocytogenes* at the rate of 2.5% [27]. Similar to our findings, Ayaz et al. (2018) found that 3.4% of cattle carcasses were contaminated with *L. monocytogenes* [28]. Biofilm formation, which takes a few hours to several days or months, is a slow process depending on the culture circumstances [29]. The attachment and biofilm-formation let surface colonization in food processing environments [30]. Predictive microbiology tools indorse the mathematical modeling of pathogen responses to diverse environmental circumstances [31]. New approaches in predictive microbiology clarify the use of substrates that contribute to the dynamics of the biofilm-formation [32]. In order to predict the biofilm-formation ability of the isolates in the study at different temperature and time parameters, phenotypic biofilm formation of *L. monocytogenes* on abiotic surfaces was basically revealed. In addition, the study was supported genotypically by revealing the virulence gene profiles that contributes to biofilm production [33–35]. Among the investigated virulence genes of 14 *L. monocytogenes* isolates obtained from the carcass, the genes with the highest prevalence were determined as *sigB* and *inlA*. The *sigB* in *L. monocytogenes* helps for viability throughout carbon starvation, and resistance to environmental stress [36]. Internalin proteins are connected to the impact on biofilm in *L. monocytogenes* as well as its hydrophobicity, motility, virulence, internalization into eukaryotes and staying alive in different milieus [37]. Also, biofilm-formation markers including *prfA*, *actA*, *inlA*, and *plcA* play a remarkable part in the survival and persistence of *L. monocytogenes* [38]. Environmental parameters such as temperature may regulate genes with regard to virulence markers or structures that result in changes in the cell surface, which may impress compliance with the nascency of flagella in *L. monocytogenes* [39].

According to the original data, the number of moderate isolates in the first seven days was higher at 4 °C than at 37 °C and 22 °C. After one week, the biofilm-forming capacity of all isolates exposed to temperatures at 4 °C, 22 °C, and 37 °C was strong. *Listeria* is of peritrichous flagella and motile at 20–25 °C but is immotile or less obviously motile at 37 °C [40]. Being a psychrotroph and able to thrive at low temperatures due to transcriptional machinery is a crucial for *L. monocytogenes* to yield infectious dose levels on contaminated refrigerated aliments [41, 42]. Uncovering the mechanisms behind this phenotypic event is essential to develop novel interventions against this pathogen in foods kept in cold milieus. Bacteria combat cold stress by reducing cell membrane fluidity and enzyme activity. Furthermore, the cold stress responses of *L. monocytogenes* appear to be essential for survival in the food matrix and crucial virulence traits. It influences biofilm-formation by mediating flagella surface adhesion [43, 44]. *L monocytogenes* in slaughterhouses and carcasses may risk for human listeriosis as *L. monocytogenes* can grow at cooling temperatures and form biofilms in slaughterhouses and meat processing plants [45].

Bonsaglia et al. (2014) indicated that biofilm production on polystyrene microplates showed that *L. monocytogenes* did not adhere well to this material, because at 4 °C, after 24 and 48 h of incubation, only 2 (6.2%) out of 32 strains produced biofilm. Bonsaglia et al. (2014) also reported that the results at 20 °C did not differ from those at 4 and 35 °C after 24 h of incubation, 2 (6.2%) strains produced biofilm, which increased to 4 (12.4%) in 48 h [46].

Kadam et al. (2013) found that the least biofilm was formed at 12 °C and the highest at 37 °C [47]. Kadam et al. (2013) and Mai and Conner (2007) suggested that the biofilm-formation rate increases with elevated temperature [47, 48]. Nilsson et al. (2011) observed that *L. monocytogenes*, showed maximum biofilm growth after 48 h of cultivation [49]. Fan et al. (2020) indicated that temperature significantly affects *Listeria monocytogenes* biofilm formation [50]. The effects of different factors on biofilm-formation are strain-dependent. Unraveling the impact and regulatory mechanisms of environmental factors on *L. monocytogenes* biofilm-formation is of value for appropriate risk assessment programs in the food industry.

In this study, the prediction performance of the original data was determined by performance measurement metrics (ME, MAE, MSE, RMSE, MPE and MAPE). ME is the average error value of the actual value. MSE calculate the variability in forecast errors. RMSE measures the average magnitude of the error. MPE is the ratio of error at a particular point of time in the series. If the MPE is negative and large enough, this forecasting method will produce a high forecast [51]. MAPE is the more accurate statistic indicator. MAPE expresses the percentage of forecast error to actual demand over a certain period, giving information on the percentage error being too high or low. It is stated that the performance of the compared models mostly depends on RMSE, MAE and MAPE [52]. According to the criteria proposed by Lewis (1982), it has been reported that the applied model produces successful high-accuracy predictions if MAPE values are below 10%. However, a value of 10–20% indicates a good estimate, 20–50% indicates a reasonable estimate, and when it is higher than 50%, it indicates a wrong estimate [53]. In the study, no MAPE value was above 50%. The 3 days to 7 days group had a reasonable prediction accuracy of over 20% for MAPE values at 37 °C, 22 °C, and 4 °C. RMSE values for the 3 days to 7 days group data at 37 °C, 22 °C, and 4 °C were also higher than other measurement groups. This could be due to less data being used in this time period. Although MAPE values were generally below 10% in measurements at 22 °C, the values were higher than 37 °C, 4 °C, and − 20 °C for the majority of the prediction groups at 22 °C. Additionally, the highest MAPE belonged to the 3 days to 7 days group at 22 °C, and the prediction accuracy was lower than measurements at other temperatures. Therefore, it can be inferred that measurements at 22 °C had lower predictive accuracy, compared to predictions from other temperatures. Among the prediction groups, MAPE values at 22 °C were close to each other, except for the days of 7 and 21 predictions. This can be attributed to the fact that motility improves biofilm stability [54].

In the prediction of the 21st and 30th days with the 15-days measurement at 37 °C, MPE values were negative and RMSE values were close to zero, and predictability was high in these groups. In the measurements after 7 days to 15 days group at 4 °C, MAPE values showed high prediction accuracy. An increase in the number of negative MPE values was observed in the group measurements at lower temperatures. When RMSE was evaluated, biofilm formation prediction accuracy of biofilm forming isolates at -20 °C was higher than at other temperatures in all prediction groups. Therefore, overall the best OD prediction accuracy belonged to the data obtained from biofilm formation at -20 °C. Freezing ceases the activities of spoilage microorganisms in and on foods [55]. An inappropriate thawing procedure gives rise to the activation and proliferation of current residing dormant microbiota on meat surface [56]. Therefore, it was deemed appropriate to examine the biofilm formation at -20 °C. This study used indicative parameters for evaluating the biofilm-forming ability to estimate the optimal temperature-time. As the predictability increases, the error metrics decreases; the highest predictability was that of the OD values indicating biofilm-forming abilities of the isolates kept at -20 °C.

For all temperatures studied, especially after the 3 days to 7 days forecast group, there was a significant decrease in the error metrics and the forecast accuracy increased. When evaluating the best prediction group, the lowest RMSE at 37 °C, 22 °C and 4 °C belonged to the 15 days to 21 days prediction group. For the OD predictions obtained at -20 °C, the 15 days to 21 days prediction group had also a good performance and the lowest RMSE belonged to the 7 days to 15 days group.

Moraes et al. (2018) reported that the models they developed and presented are sufficient for the evaluating of *S. enterica* adhesion and biofilm-formation on stainless steel surfaces. Moraes et al. (2018) also stated that in most cases the differences between predictions and observations were due to false positives (adhesion/no biofilm) [57]. Adamczewski et al. (2022) estimated the count of *L. monocytogenes* in butter by the mathematical approach and found the tool useful [58]. Predictive data-mining tools are designed to help us understand what the useful information looks like and what has happened during past procedures. Time series forecasting models have advantages for public health policy applications [59]. ARIMA-based modeling become a standard tool for time series and simple enough to be widely understood and thus, it could be integrated into microbial growth-survival fields [60]. One of the genuine aspects of this study is that it considers the evaluation the prediction of biofilm-formation on abiotic surfaces by exposure of *L. monocytogenes* isolates to various times and temperatures using the ARIMA model. Overall, our findings show that the ARIMA model has high performance in predicting biofilm formation of *L. monocytogenes* isolates at all tested temperatures and time parameters.

In conclusion, this study will guide in using indicator parameters to evaluate biofilm forming ability to predict optimum temperature-time. The models integrated with this study can be useful tools for industrial application and risk assessment studies using different parameters such as pH, NaCl concentration, and temperature applied during food processing and storage on the biofilm-formation ability of *L. monocytogenes* and other pathogens. Thus, this model approach is crucial as it can provide the basis for implementing more effective hygienic procedures to protect public health.

### Electronic supplementary material

Below is the link to the electronic supplementary material.

Supplementary Material 1

## Data Availability

All data collected or analyzed during this study are included in this manuscript.
