# Supplementary material for: The ARIMA model approach for the biofilm-forming capacity prediction of Listeria monocytogenes recovered from carcasses
Source: BMC Vet Res. 2024 Mar 27;20:123. doi: 10.1186/s12917-024-03950-y (PMC10967039; doi:10.1186/s12917-024-03950-y)
Supplement: Supplementary file 1 — Supplementary Material 1 [file 12917_2024_3950_MOESM1_ESM.docx]

**Supplementary Material**

**Table 1.** The Virulence primer pairs used for the present study

| Target gene | Nucleotide Sequence | References |
| --- | --- | --- |
| *plcA*-f | CTAGAAGCAGGAATACGGTACA | Rantsiou et al, 2012 |
| *plcA*-r | ATTGAGTAATCGTTTCTAAT |  |
| *plcB*-f | CAGGCTACCACTGTGCATATGAA | Olesen et al, 2009 |
| *plcB*-r | CCATGTCTTCYGTTGCTTGATAATTG |  |
| *hly*-f | TACATTAGTGGAAAGATGG | Rantsiou et al, 2012 |
| *hly*-r | ACATTCAAGCTATTATTTACA |  |
| *sigB*-f | CCAAGAAAATGGCGATCAAGAC | Rantsiou et al, 2012 |
| *sigB*-r | CGTTGCATCATATCTTCTAATAGCT |  |
| *inlA*-f | AATGCTCAGGCAGCTACAMTTACA | Olesen et al, 2009 |
| *inlA*-f | CGTGTCTGTTACRTTCGTTTTTCC |  |
| *inlB*-f | AAGCAMGATTTCATGGGAGAGT | Olesen et al, 2009 |
| *inlB*-r | TTACCGTTCCATCAACATCATAACTT |  |
| *inlC*-f | ACTGGTCAGAAATGTGTGAATGA | Hadjilouka et al, 2016 |
| *inlC*-r | CCATCTGGGTCTTTGACAGT |  |
| *inlJ*-f | TGCGTAAATGCTCACATCCAAG | Hadjilouka et al, 2016 |
| *inlJ*-r | TTGCCCTTCAGCATCCAAGT |  |

**Table 2.** Virulence characteristics of *Listeria monocytogenes*

| Isolates codes | Virulence factors | | | | | | | |
| --- | --- | --- | --- | --- | --- | --- | --- | --- |
|  | *hlyA* | *sigB* | *plcA* | *plcB* | *inlA* | *inlB* | *inlC* | *inlJ* |
| 124 | - | + | - | - | + | + | + | - |
| 200 | + | - | - | + | - | - | - | - |
| 207 | - | - | - | - | - | - | + | - |
| 208 | - | - | - | - | - | - | + | - |
| 217 | + | - | - | + | - | - | + | - |
| 226 | - | + | - | + | + | + | - | - |
| 300 | - | - | - | - | + | - | - | - |
| 302 | - | + | - | - | + | - | - | - |
| 309 | - | + | - | - | + | - | - | - |
| 326 | - | + | - | - | + | - | - | + |
| 348 | - | + | - | - | - | - | - | - |
| 352 | - | + | - | - | + | - | - | + |
| 360 | - | - | - | - | - | - | - | + |
| 377 | - | + | - | - | + | - | - | + |
| Total | 2 | 10 | 0 | 3 | 10 | 2 | 4 | 5 |
| Percentage | 14.2 | 71.4 | 0 | 21.4 | 71.4 | 14.2 | 28.4 | 35.7 |
